# Quantitative Assessment of Mammary Gland Density in Rodents Using Digital Image Analysis

**DOI:** 10.1186/1480-9222-13-4

**Published:** 2011-06-10

**Authors:** John N McGinley, Henry J Thompson

**Affiliations:** 1 Cancer Prevention Laboratory, Colorado State University, 1173 Campus Delivery, Fort Collins, CO 80523, USA

## Abstract

**Background:**

Rodent models have been used extensively to study mammary gland development and for studies of toxicology and carcinogenesis. Mammary gland gross morphology can visualized via the excision of intact mammary gland chains following fixation and staining with carmine using a tissue preparation referred to as a whole mount. Methods are described for the automated collection of digital images from an entire mammary gland whole mount and for the interrogation of digital data using a "masking" technique available with Image-Pro^® ^plus image analysis software (Mediacybernetics. Silver Spring, MD).

**Results:**

Parallel to mammographic analysis in humans, measurements of rodent mammary gland density were derived from area-based or volume-based algorithms and included: total circumscribed mammary fat pad mass, mammary epithelial mass, and epithelium-free fat pad mass. These values permitted estimation of absolute mass of mammary epithelium as well as breast density. The biological plausibility of these measurements was evaluated in mammary whole mounts from rats and mice. During mammary gland development, absolute epithelial mass increased linearly without significant changes in mammographic density. Treatment of rodents with tamoxifen, 9-cis-retinoic acid, or ovariectomy, and occurrence of diet induced obesity decreased both absolute epithelial mass and mammographic density. The area and volumetric methods gave similar results.

**Conclusions:**

Digital image analysis can be used for screening agents for potential impact on reproductive toxicity or carcinogenesis as well as for mechanistic studies, particularly for cumulative effects on mammary epithelial mass as well as translational studies of mechanisms that explain the relationship between epithelial mass and cancer risk.

## Background

Rodent models have been used extensively to study mammary gland development and for investigations of many aspects of reproductive toxicology and breast carcinogenesis [1,2]. Mammary gland development occurs postnatally and is affected by initiation of ovarian function as occurs in humans at the time of menarche [3]. The development of the rodent mammary gland has been described in detail [3-5]. However, while representative images of mammary gland gross morphology are frequently reported in the literature, the actual quantification of changes in gross morphology is uncommon and rarely have such analyses been extended to the entire mammary gland rather than a small representative area. The work reported herein describes a technique for automated collection of digital images of rodent mammary glands excised as a mammary gland chain. While the mammary glands of rodents occur symmetrically in pairs and are divided into those that occur in the cervical-thoracic region and the abdominal-inguinal region, visualization of the cervical-thoracic glands in either the rat or the mouse is hindered by a band of muscle that lies interspersed between the glands. The muscle is difficult to uniformly remove and thus renders excised chains impossible to digitally imagine without confounding of results due to residual adhering muscle. However, no muscle bands are present in the abdominal-inguinal mammary gland chain of either species and for that reason the abdominal-inguinal chain is traditionally imaged by investigators. The abdominal-inguinal mammary gland chain is the focus of the work reported.

**Figure 1:**
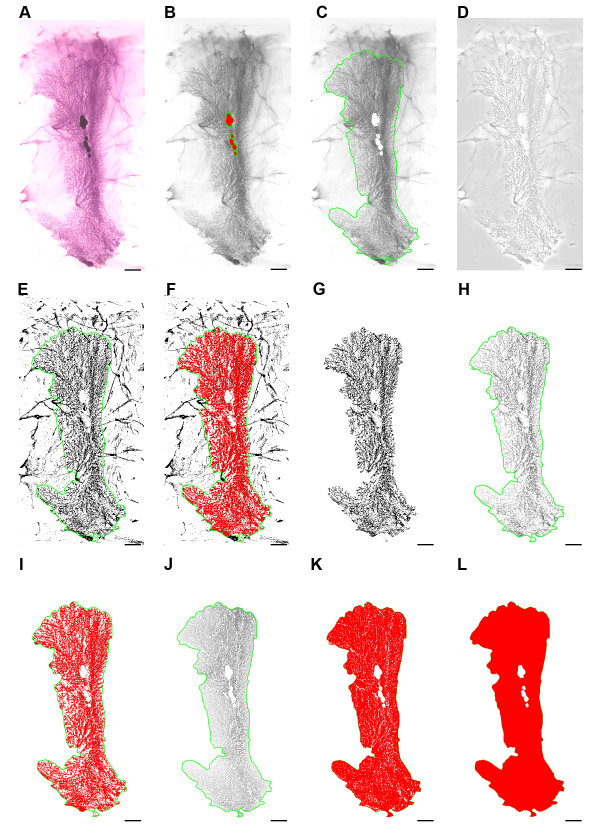
**Stepwise example of whole mount digital image analysis**. (A) original color image, (B) image converted to gray scale and lymph nodes marked, (C) lymph nodes excised and epithelia circumscribed as an AOI (green). (D) flattened image, (E) high contrast enhancement applied, (F) setting image threshold, (G) inverted digital mask, (H) extracted epithelia (I) measured epithelia (J) extracted interductal fat pad (K) measured interductal fat pad, (L) total measurement. Bars = 0.5 cm.

Mammary gland morphology is studied indirectly in women via mammography. Other than the use of mammography for the early detection of small pathologies that might otherwise be missed during a physical exam, mammograms are also used to estimate breast density, i.e. the ratio of radiodense fibroglandular breast tissue to the total amount of breast tissue present; non glandular, radiolucent breast tissue is also determined. Breast density is an independent risk factor for breast cancer [6]. In reality there are four methods for estimating breast density in humans[7], but the two methods that use computer-assisted image analysis to determine area or volume of glandular tissue are parallel to the digital analyses reported herein which also measure mammary epithelial mass in absolute terms or expressed as a percent area or percent volume. The similarities in methods are remarkable. In the human, the total area or volume of the breast is determined by the physical contour of the breast which can be reproducibly demarcated. In the rodent the contour of the mammary fat pad is operationally defined via a line circumscribed around the most distal end buds of the mammary gland chain (Figure 1).

## Methods

Abdominal inguinal mammary gland chains were excised at necropsy and prepared as whole mounts as described by Thompson et al [8]. The work followed ethical guidelines approved by the Colorado State University Animal Care and Use Committee. The left chain was fixed in methacarn (24 hrs) and the right chain fixed in 10% neutral buffered formalin (24 hrs). Fixed mammary whole mounts were dehydrated using a series of graded ethanols, cleared in xylene, hydrated using a series of graded ethanols and stained in modified Mayer's carmalum (0.4% carmine in 1.0% aluminum potassium sulfate) for 3 days. Whole mounts were rinsed in tap water to remove excess carmine stain, dehydrated using a series of graded ethanols and cleared in xylene.

Cleared whole mounts were prepared using a modified protocol described by Wellings et al [9]. Whole mounts were placed in a 4 × 6 inch 4.5 mil thick kapak^® ^(3 M, St. Paul, MN) heat seal bags, filled with 20 ml of methyl salicylate (Sigma. St Louis, MO) and the bags crimped at the top using a kapak^® ^(3 M) heat sealer. All alcohols and solvents were used under a chemical fume hood. In addition, a NIOSH approved air-purifying unit (3 M) was worn during dispensing and handling of methyl salicylate to avoid unnecessary exposure.

Air bubbles were removed by placing the bagged whole mount on a flat surface and forcing the air bubbles to the periphery of the whole mount using slight finger pressure. The bagged whole mounts were placed vertically in the heat sealer. Residual air and excess methyl salicylate was displaced to the top by applying slight pressure to the bottom half of the bag containing the whole mount. The bag was crimped in the middle with the whole mount in the bottom of the bag and the residual air and excess methyl salicylate at the top. The bag was cut along the midline to separate the two halves.

Digital images of the mammary gland whole mounts were captured using a semi-automated image acquisition system (North Central Instruments, Plymouth, MN). The components of this system included a 3.0 megapixel CMOS digital camera (Clemex Technologies, Inc. Longueuil, Canada) mounted on a Leica Z16 APO monocular zoom lens 16:1 with a magnification range of 0.57 - 9.2x. The camera and lens were mounted on a Leica Z motor attached to a transmitted light base with a 100 × 100 mm motorized stage (Clemex Technologies, Inc.). An X-Y control box and joystick (Clemex Technologies, Inc.) in conjunction with a Pentium 4 desktop PC (Dell, Round Rock, TX) and Captiva v4.0 software (Clemex Technologies, Inc.) were used for image capture.

Whole mounts were placed on a 6 mm thick sheet of white acrylic plastic (Gagne, Inc. Johnson City, NY) mounted on top of the motorized stage to act as a diffuser. Specimens were trans-illuminated using a 20 V/150 W halogen lamp light source (Volpi, Auburn, NY) with daylight filter mounted at the rear of the base. A series of Z-stack images were automatically captured at 10&3215; magnification using the motorized stage in conjunction with the Captiva 4.0 software (Clemex Technologies, Inc.) and × Y controller. The software seamlessly merged tiled Z-stack images together to form a single uniformly focused composite image based on a best contrast algorithm. Resulting images were saved as TIF files.

Image-Pro^® ^plus 4.5 (Mediacybernetics. Silver Spring, MD) image analysis software was used to quantify images according to a modified version of a protocol described by Thompson et al [10]. A composite image of a stage micrometer was captured under the same conditions as the whole mounts. This image was used to calibrate the area measurements in units of cm^2^. A standard optical density curve relating gray level (0-255) to intensity (0 - 2.4) was used to measure ΣOD. An Image Pro macro was written to simplify image analysis. This not only increased the speed of analysis, but also eliminated potential errors.

The macro converted the original color images (Figure 1A) to 8 bit gray scale. Lymph nodes were selected using the irregular area of interest (AOI) tool wand option (Figure 1B). A mask of the selected lymph node areas was created and an "or" image operation process was applied to extract the lymph nodes from the gray scale images. Mammary gland epithelium was manually circumscribed using the irregular AOI tool trace option (green outline). Folds, wrinkles, residual muscle and nipples were excluded from the AOI (Figure 1C). The AOI was removed and the image was "flattened", a digital filtering process that decreases the variation in the intensity of background pixels, which is essential when attempting to properly threshold areas within an image that contain similar intensities, e.g. differentiating mammary epithelia from interductal fat pad within a whole mount (Figure 1D). High contrast was applied to the flattened image to enhance visualization of dense areas and the AOI was reapplied (Figure 1E). A 0-254 gray level selection threshold, marked in red was applied to the AOI (Figure 1F), from which two binary masks were created, one mask containing non-dense elements (interductal fat pad) within the AOI and other inverted mask containing dense elements (mammary epithelia) within the whole mount AOI (Figure 1G)

An "or" image operation was performed using the gray scale image (Figure 1C) and the inverted dense area mask (Figure 1G) to extract only the dense areas (mammary epithelia) from the whole mount (Figure 1H), from which area and volume estimates of the mammary epithelia were obtained (Figure 1I). A similar image process was applied to the gray scale image (Figure 1C) and non-dense area mask to extract only non-dense areas (interductal fat pad) from the whole mount (Figure 1J), which in turn was used to estimate area and volume of the interductal fat pad (Figure 1K). The circumscribed AOI in the original gray scale image (Figure 1C) was then used to obtain total area and volume estimates, thus representing combined epithelial and interductal fad pad measurements of the mammary chain (Figure 1L). All measurements were exported to an Excel spreadsheet (Microsoft. Redmond, WA) via dynamic data exchange. Data were then imported into Systat 13 (Systat Software, Inc., Chicago, IL) for statistical analysis.

## Results and discussion

The intent of the digital imaging technique described in the Methods section is to provide a tool to quantify, modify, and investigate mechanistically mammary epithelial mass and density. The work reported herein is a critical first step in achieving that objective and closely models the methods used to quantify breast density in women [11]. In the following sections, the digital image analysis tool for rodent mammary gland was applied to a number of biological contexts to illustrate usage and to evaluate the biological plausibility of what is measured. For each example to which the analysis tool was applied, potential research applications are discussed.

### Mammary gland development

**Figure 2:**
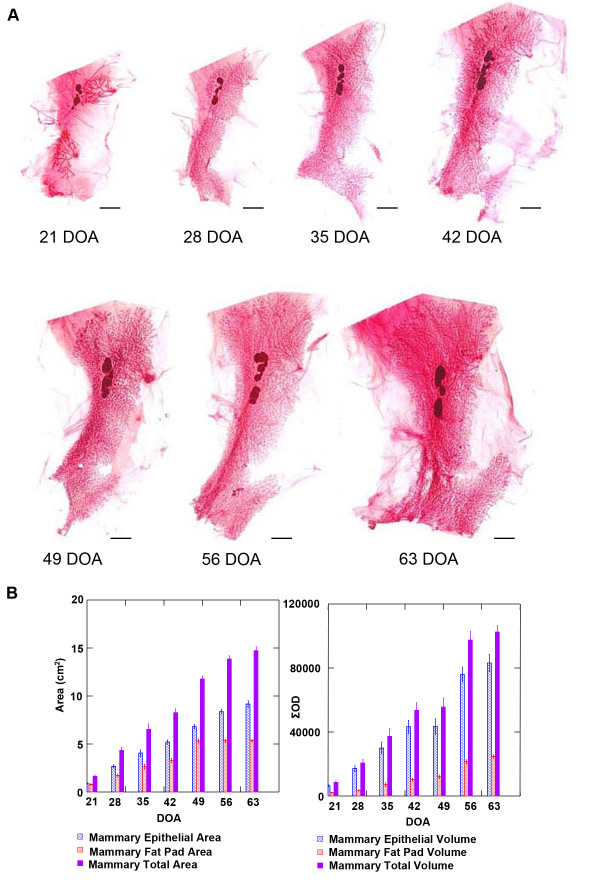
**Time course of rat mammary gland whole mounts**. (A) Stained whole mounts ranging from 21 to 63 days of age (DOA), bars = 0.5 cm. (B) Area and volume measurements.

**Figure 3:**
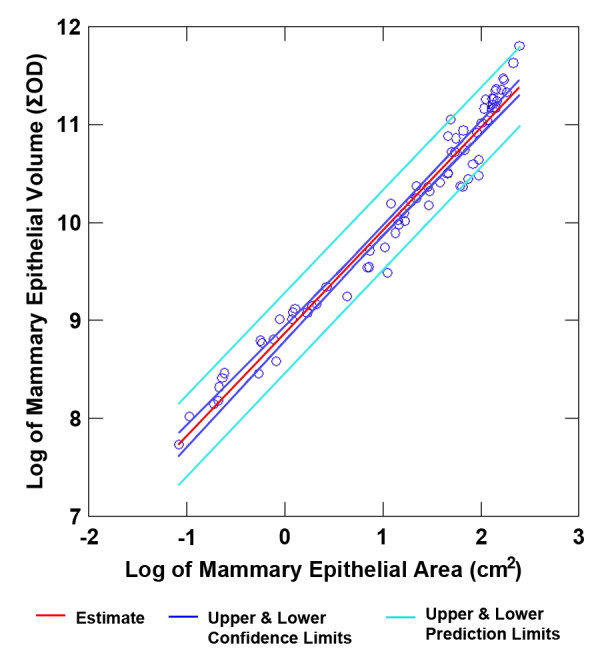
**log transformed regression analysis of mammary epithelial area and volume**.

The digital analysis tool was first applied to the development of the mammary gland which occurs postnatally in mammals [3,4]. Female Sprague-Dawley rats were euthanized at 21, 28, 35, 42, 49, 56 and 63 days of age (DOA), *n *= 9/time point. Figure 2 shows representative mammary gland images at 7 day intervals for this time course. The hypothesis underlying this experiment was that the absolute amount of mammary epithelium in the abdominal-inguinal chain would increase over the timeframe of the experiment. The amount of mammary epithelium was quantified using either an area algorithm or a volume algorithm as is currently done in the analysis of digital images of human mammograms [11-13]. As shown in Table 1, the measurements made in humans, total fibroglandular mass, mass of non-glandular tissue, total breast mass, and breast density (fibroglandular mass/total breast mass), estimated by the area or volumetric method, have direct counterparts in the analysis of rodent tissue. As shown in Figure 3, there was excellent agreement between these two methods of estimating absolute mass of mammary epithelium, with 95% of the variance in the volume measurement being accounted for in the measurement of area (r^2 ^= 0.95, *p *< 0.001 on the log transformed data). For this reason, the focus of this analysis and the others reported in this paper was on the area algorithm since area estimates have been reported to be more useful in the clinical setting [13]. Total mammary epithelial mass (*p *< 0.001) increased with increasing age; whereas, the area associated with the interductal fat pad component of the gland increased from 21 to 49 DOA, but growth rate was reduced after 49 DOA, perhaps indicting a slowing of ductal extension while ductal branching increased. These findings are consistent with the occurrence of sexual maturation which is manifest as vaginal opening between 30 and 38 DOA in Sprague Dawley rats and the occurrence of the first estrous cycle which occurs between 40 and 45 DOA [2]. Interestingly, mammary density did not change markedly between 35 and 63 DOA, perhaps due to the limitations in determining total breast volume as mentioned in the Introduction section. These data provide evidence that the method of digital analysis presented herein is quantitative and the absolute epithelial mass data meet the criteria of biological plausibility.

**Table 1:** Area Measurements of Time Course Mammary Gland Whole Mounts

DOA	Epithelial area (cm^2^)	Interductal fat pad area (cm^2^)	Total area (cm^2^)	Epithelial density area %	Epithelial volume (∑OD)	Interductal fat pad volume (∑OD)	Total volume (∑OD)	Epithelial density volume %
21	0.86 ± 0.08^a^	0.81 ± 0.07^a^	1.62 ± 0.14^a^	52.68 ± 1.27^a^	6425.5 ± 583.8^a,h^	2164.0 ± 211.5^a,h^	8570.9 ± 779.2^a,h^	75.07 ± 0.83^a,h^
28	2.69 ± 0.16^b^	1.73 ± 0.15^b^	4.36 ± 0.28^b^	61.91 ± 1.00^b,h^	17225.8 ± 1744.7^b,h,i^	3460.6 ± 458.4^b,h,i^	20700.6 ± 2168.1^b,h,i^	83.47 ± 0.84^b,i^
35	4.07 ± 0.31^c^	2.63 ± 0.25^c,h^	6.53 ± 0.54^c^	62.59 ± 0.63^c,h^	30042.1 ± 3548.6^c,i,j,k^	7178.2 ± 1147.3^c,i,j^	37220.3 ± 4692.7^c,i,j,k^	81.17 ± 0.51^c,i^
42	5.20 ± 0.21^d^	3.28 ± 0.18^d,h^	8.28 ± 0.37^d^	62.89 ± 0.73^d,h^	43344.1 ± 3761.8^d,j,l^	10286.4 ± 716.4^d,j,k^	53630.6 ± 4388.7^d,j,l^	80.63 ± 0.71^d,i^
49	6.82 ± 0.20^e^	5.32 ± 0.20^e,i^	11.77 ± 0.32^e^	58.00 ± 1.04^e,h^	43475.6 ± 4520.7^e,k,l^	12114.0 ± 1031.7^e,k^	55589.6 ± 5449.1^e,k,l^	77.90 ± 0.94^e,h,i^
56	8.38 ± 0.23^f,h^	5.35 ± 0.15^f,i,j^	13.85 ± 0.35^f,h^	60.50 ± 0.58^f,h^	76073.4 ± 4138.3^f,m^	21564.5 ± 1122.4^f,l^	97637.8 ± 4899.8^f,m^	77.83 ± 0.74^f,h,i^
63	9.19 ± 0.32^g,h^	5.34 ± 0.09^g,j^	14.74 ± 0.38^g,h^	62.23 ± 0.66^g,h^	88775.5 ± 7127.8^g,m^	24786.9 ± 1160.2^g,l^	113562.4 ± 8120.4^g,m^	77.85 ± 0.75^g,h,i^

Values are means ± SEM (*n *= 9 per DOA). Values in a column with different superscripts are statistically different, *p *< 0.05 by method of Bonferroni for multiple comparisons.

From a research perspective, these data indicate that this technique could be used to quantify the net cumulative effects of an intervention over time on mammary epithelial mass, i.e. the net balance between cell proliferation and cell death, as well as how changes in mass of the non-epithelial component of the mammary fat pad correspond to the changes in mass that occur in the epithelial compartment. In this context, the measurement of epithelial density would reflect the complexity of the growth pattern that was occurring.

### Effect of endocrine manipulations

**Figure 4:**
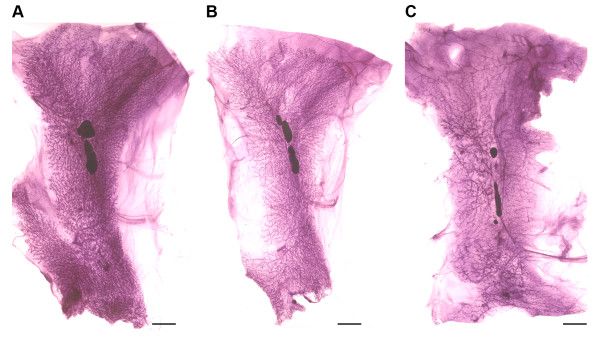
**Rat mammary gland whole mounts**. (A) Control, (B) Tamoxifen (1 mg), (C) Ovex. Bars = 0.5 cm.

In order to further evaluate biological plausibility, two experiments were conducted. In the first experiment, mature female Sprague Dawley rats were bilaterally ovariectomized at 110 DOA. Following ovariectomy, the rats were maintained for an additional 5 weeks under standard housing conditions. The hypothesis tested was that mammary gland mass would be reduced in comparison to rats whose ovaries had not been removed (intact animals). Representative images from intact and ovariectomized rats are shown in Figure 4. As shown in Table 2, total mammary epithelial mass was reduced by 33% in ovariectomized rats, (8.14 vs 5.48, *p *= 0.002). The total area in which the mammary gland was encompassed was also reduced, consistent with a reduction in the overall size of the gland (15.34 vs 11.05, 28% decrease, *p *< 0.001). However when epithelial mass was divided by mass of the entire gland, to estimate breast density, a 6.6% reduction in density was observed, but the decrease was not statistically significant (52.9 vs 49.4, *p *= 0.177). This suggests that during this short term loss of ovarian hormone function in the mature rat that the amount of mammary epithelium was decreasing at a more rapid rate than epithelial complexity measured as mammary epithelium mass per square centimeter of mammary gland.

**Table 2:** Mammary gland density analysis of ovariectomized rats

Treatment	Epithelial area (cm^2^)	Interductal fat pad area (cm^2^)	Total mammary gland area (cm^2^)	Epithelial density area %
Control (n = 5)	8.15 ± 0.99	7.20 ± 0.38	15.34 ± 0.77	52.9 ± 4.0
Ovariectomy (n = 5)	5.48 ± 0.71	5.57 ± 0.28	11.05 ± 0.77	49.4 ± 3.5
*p*	0.002	< 0.001	< 0.001	0.177

Values are means ± SD

In the second experiment, female Sprague Dawley rats were administered 1 mg tamoxifen as tamoxifen citrate in a purified diet beginning at 28 DOA. Rats were treated for 6 weeks and then euthanized. This dose of tamoxifen is highly protective against chemically induced mammary carcinogenesis under these conditions [14]. As summarized in Table 3, tamoxifen treated rats had a 46.5% reduction in mammary epithelial mass (8.14 vs 4.37, *p *< 0.001) and a 24.3% reduction in total area (15.34 vs 11.06. *p *< 0.001. Unlike the effects of ovariectomy, treatment with tamoxifen in this content reduced breast density from 52.9% vs 39.5%, *p *< 0.001, an indication that the complexity of the mammary gland was reduced by tamoxifen. Both the reduction in mammary gland mass and in mammary gland density are consistent with these parameters being predictive of cancer risk as proposed for breast density in human populations. The comparison between the effects of ovariectomy and tamoxifen, both of which are protective against the occurrence of breast cancer show that it is possible to have a reduction in mammary epithelial mass with or without a reduction in density based on our operational definition of determining mass of the entire mammary gland. From a research perspective, these findings indicate that the digital analysis of epithelial density may be of particular value in elucidating the relationship between breast density and cancer risk as well as serve a useful purpose in screening compounds for reproductive toxicity, a good example of which would be environmentally occurring neuroendocrine disrupters [15,16].

**Table 3:** Mammary gland density analysis of tamoxifen treated rats

Treatment	Epithelial area (cm^2^)	Interductal fat pad area (cm^2^)	Total mammary gland area (cm^2^)	Epithelial density area %
Control (n = 13)	8.07 ± 0.91	6.98 ± 0.99	15.06 ± 1.51	53.7 ± 4.1
Tamoxifen (n = 23)	5.77 ± 0.72	6.76 ± 1.08	12.53 ± 1.64	46.2 ± 3.2
*p*	< 0.001	0.538	< 0.001	< 0.001

Values are means ± SD

### Effect of 9-cis Retinoic Acid

**Figure 5:**
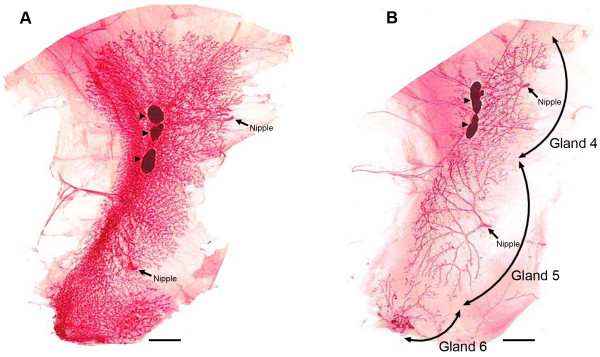
**Rat mammary gland whole mounts**. (A) Control, (B) 9-cis Retenoic acid (120 ppm). Bars = 0.5 cm

Dramatic effects in mammary gland development can be seen in young animals treated with agents such as 9-cis retinoic acid (Figure 5). Female Sprague Dawley rats were obtained at 21 DOA and treated with 120 mg 9-cis retinoic acid per kg purified diet (AIN 93-G). Feeding experimental diets was initiated at 28 DOA and continued for 6 weeks at which time rats were euthanized. While it is clear from Figure 5 that mammary gland size and complexity were reduced by treatment, the quantification of this effect is shown in Table 4. Epithelial mass (5.442 vs 1.667, *p *< 0.001) was reduced by 69.4% by treatment with 9-cis retinoic acid; whereas total mammary gland size was 47.6% lower (9.664 vs 5.064, *p *< 0.001). When the percent mammary gland density was computed, it was reduced by 41.7% (56.3 vs 32.8, *p *< 0.001). This provides yet another perspective from which to evaluate biological plausibility of the measurement technique. From a research perspective, it is well known that this retinoid is a strong chemopreventive agent in the mammary gland at this dietary concentration [17,18]. This raises the possibility that the digital assessment tool could be used to screen agents for chemopreventive activity as well as to study mechanisms at the whole tissue level of complexity.

**Table 4:** Mammary gland density analysis of 9-cis retinoic acid (120ppm) treated rats

Treatment	Epithelial area (cm^2^)	Interductal fat pad area (cm^2^)	Total mammary gland area (cm^2^)	Epithelial density area %
Control (n = 5)	5.44 ± 0.46	4.22 ± 0.33	9.66 ± 0.55	56.3 ± 2.6
9-cisRA (n = 5)	1.67 ± 0.34	3.40 ± 0.50	5.06 ± 0.79	32.8 ± 2.9
*p*	< 0.001	0.015	< 0.001	< 0.001

Values are means ± SD

### Effect of obesity

**Figure 6:**
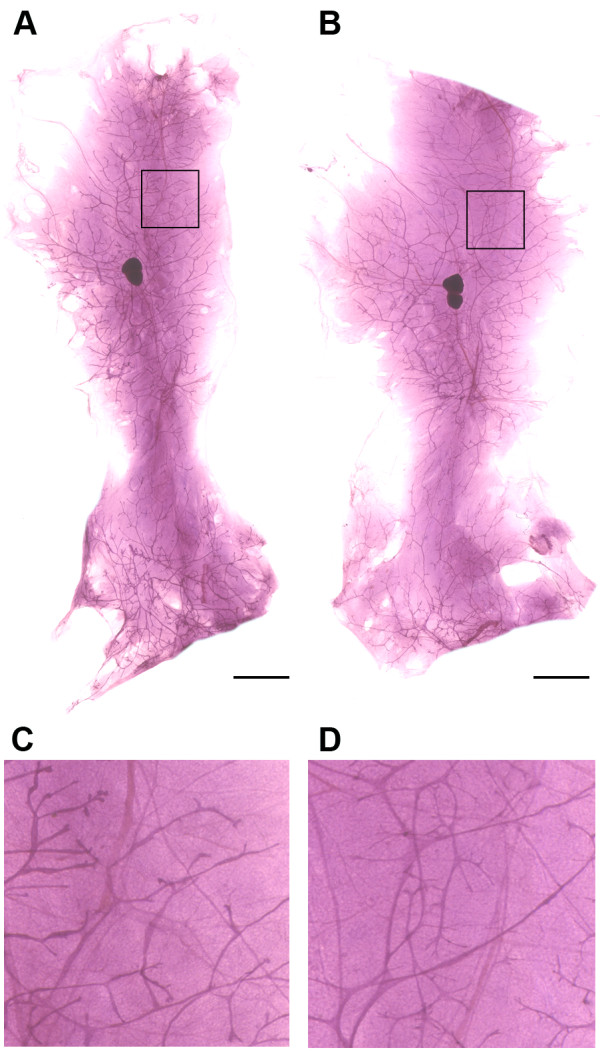
**Mouse mammary gland whole mounts**. (A) low fat diet, (B) high fat diet. (C) high resolution view of 0.5 cm^2 ^area depicted in panel A (black square). (D) high resolution view of 0.5 cm^2 ^area depicted in panel B (black square). Bars = 0.5 cm.

There is considerable interest in determining how obesity affects breast cancer risk. In humans, obesity and increased breast density are both positively correlated with breast cancer risk but inversely correlated with one another [13]. The digital analysis procedure was applied to obese mice to determine how the rodent compares with the human and to illustrate the use of the technique in the mouse. C57Bl/6 mice are known to be prone to dietary induced obesity [19,20]. Female mice were obtained from Jackson Laboratory at 21 DOA and maintained on either a non obesogenic low fat diet formulation (Research Diets D12329) or a high fat obesogenic diet (Research Diets D12331). Diets were fed for 270 days at which time mice were euthanized and mammary glands processed for analysis. Representative whole mounts from a non-obese and obese mouse are shown in Figure 6 and the effects of obesity on mammary epithelial mass and mammary epithelial density are shown in Table 5. Consistent with other reports, the body weights of the obese mice were markedly higher than that of their non-obese counterparts. Mammary epithelial mass was reduced in obese mice by 20.3% (1.58 vs 1.98, *p *= 0.259) but total area was unaffected (5.161 vs 5.247, *p *= 0.908). Mammary epithelial density was reduced by 20.5% (29.9 vs 37.6, *p *= 0.046), a finding consistent with reports that obesity is associated with a reduction in breast density in women [13]. From a research perspective, this technique affords the opportunity to address numerous research issues. Not only can questions about the seemingly contradictory roles of obesity and breast density on cancer risk be investigated mechanistically, but the technical capability now exists to quantify the cumulative effects of various engineered genotypes on mammary epithelial mass and complexity and to relate those effects to the development of cancer.

**Table 5:** Mammary gland density analysis of obese mice

Treatment	Epithelial area (cm^2^)	Interductal fat pad area (cm^2^)	Total mammary gland area (cm^2^)	Epithelial density area %
Low fat (n = 4)	1.98 ± 0.33	3.27 ± 0.23	5.25 ± 0.14	37.65 ± 5.69
High fat (n = 7)	1.58 ± 0.61	3.58 ± 0.85	5.16 ± 1.40	29.87 ± 5.20
*p*	0.259	0.492	0.908	0.046

Values are means ± SD

### General Comments

Just as there are numerous procedures and quality assurance protocols that are required for the meaningful interpretation of human mammograms [11], it is essential that rodent mammary gland chains be properly excised and spread out so that the digital images and flattening algorithms used in image analysis are subject to a low level of artifacts. The volumetric determinations are more prone to errors associated with lack of adequate spreading of the mammary gland chain when it is excised and to issues inherent in defatting and staining a thick, fatty tissue like the rodent mammary gland. The major errors in the measurement of area are associated with either under or overspreading of the mammary gland during whole mount preparation, but it is our experience that after training and practice, these errors are minimal and easily identified by data analysis for statistical outliers. While error rates appear to be lower for the area measurements, the volumetric approach offers more information but is probably best used by experienced practitioners. It is also worth noting that methacarn fixed whole mounts stained better, cleared better and had fewer problems associated with tissue detachment from the slides compared to formalin fixed counterparts.

### Limitations

As noted in the Introduction section, whereas in the human, the total area or volume of the breast is determined by the physical contour of the breast which can be reproducibly demarcated, in the rodent, the contour of the mammary fat pad is operationally defined via a line circumscribed around the most distal end buds of the mammary gland chain. Clearly, this approach underestimates total mammary gland fat pad area, but as shown in the Results and Discussion section, this biases against finding intervention effects and thus provides the basis for a robust assessment tool. Another potential limitation is that stroma is a major contributor to mammographic density in humans but appears to be substantially less conspicuous in the rodent mammary gland. In addition, while mammographic density in humans can be monitored over time, the approach described here can only be applied to excised mammary glands.

## Conclusions

The mammary gland digital analysis tool described in this paper provides a means to characterize the effects of various genetic manipulations on mammary gland morphology as well as the effects of environmental contaminants such as neuroendocrine disruptors and their associated reproductive toxicities. The approach also has potential for the preclinical investigator to conduct mechanistic studies that have direct translational value to the clinic. Many questions exist not only about the most effective manner in which to evaluate and interpret mammographic density in the clinic but also about the mechanisms that account for the predictive value of breast density for cancer risk and for explanations of complex issues such as changes in breast density and breast cancer risk with aging and the basis for the effects of adiposity and breast density on breast cancer risk.

## List of abbreviations used

AOI: Area of Interest; DOA: days of age;

## Competing interests

The authors declare that they have no competing interests.

## Authors' contributions

JNM participated in execution of the study and was responsible for preparation and image analysis of all whole mount specimens. HJT designed the study and performed the statistical analysis. All authors contributed to writing the manuscript. All authors read and approved the final manuscript.
